# Rectus Sheath Hematoma Causing Bladder Outlet Obstruction

**DOI:** 10.7759/cureus.7974

**Published:** 2020-05-05

**Authors:** Anupam K Gupta, Barbara M Parker, Andrew S Ross

**Affiliations:** 1 Surgery, Charles E. Schmidt College of Medicine, Florida Atlantic University, Boca Raton, USA; 2 Clinical Pharmacy, AdventHealth Orlando & Rockledge Regional Medical Center, Orlando, USA; 3 General Surgery, Boca Raton Regional Hospital, Boca Raton, USA

**Keywords:** rectus sheath hematoma, urinary retention, pelvic hematoma

## Abstract

A 93-year-old woman on Coumadin with history of atrial fibrillation and chronic obstructive pulmonary disease (COPD) presented with urinary retention for one day. Computed tomography (CT) of abdomen and pelvis demonstrated grade 3 rectus sheath hematoma (RSH), with the hematoma dissecting between the transversalis fascia and muscle into the prevesical space. The large-sized hematoma caused compression at the bladder outflow tract causing urinary retention. In view of age and the patient being a poor surgical candidate, the patient was managed by percutaneous drain of the hematoma to reduce size to relieve urinary symptoms. The hematoma shrunk in size over the period of next few weeks and thereby avoided surgical intervention.

## Introduction

Rectus sheath hematoma (RSH) is an unusual condition characterized by bleeding and hematoma formation between the layers of the rectus sheath [1]. As anticoagulation is becoming more prevalent, even minor trauma can precipitate RSH [1-4]. If RSH is small and present, it usually is accompanied by an abdominal bulge which can be painful [3-5]. Some can dissect into the pelvis, and if large enough, can cause pressure symptoms [5-11]. It is unusual for them to cause bladder outlet obstruction.

## Case presentation

**Figure 1:**
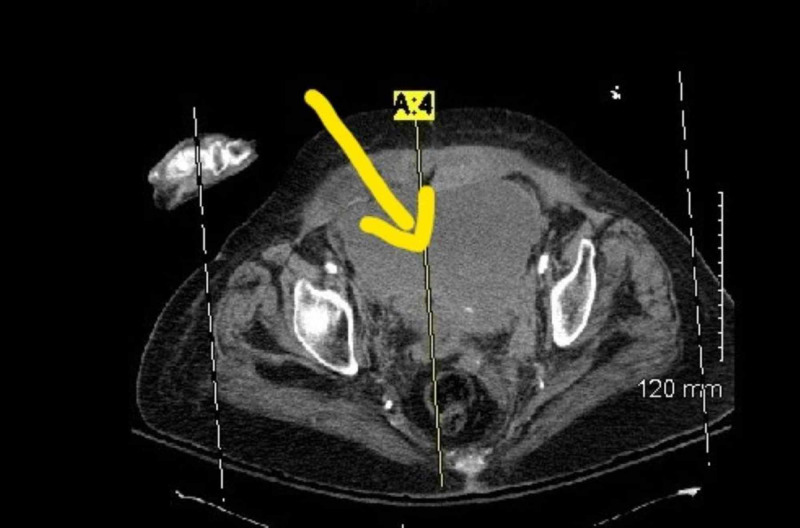
Rectus sheath hematoma below the arcuate line in the pelvis compressing the bladder with no blush

**Figure 2:**
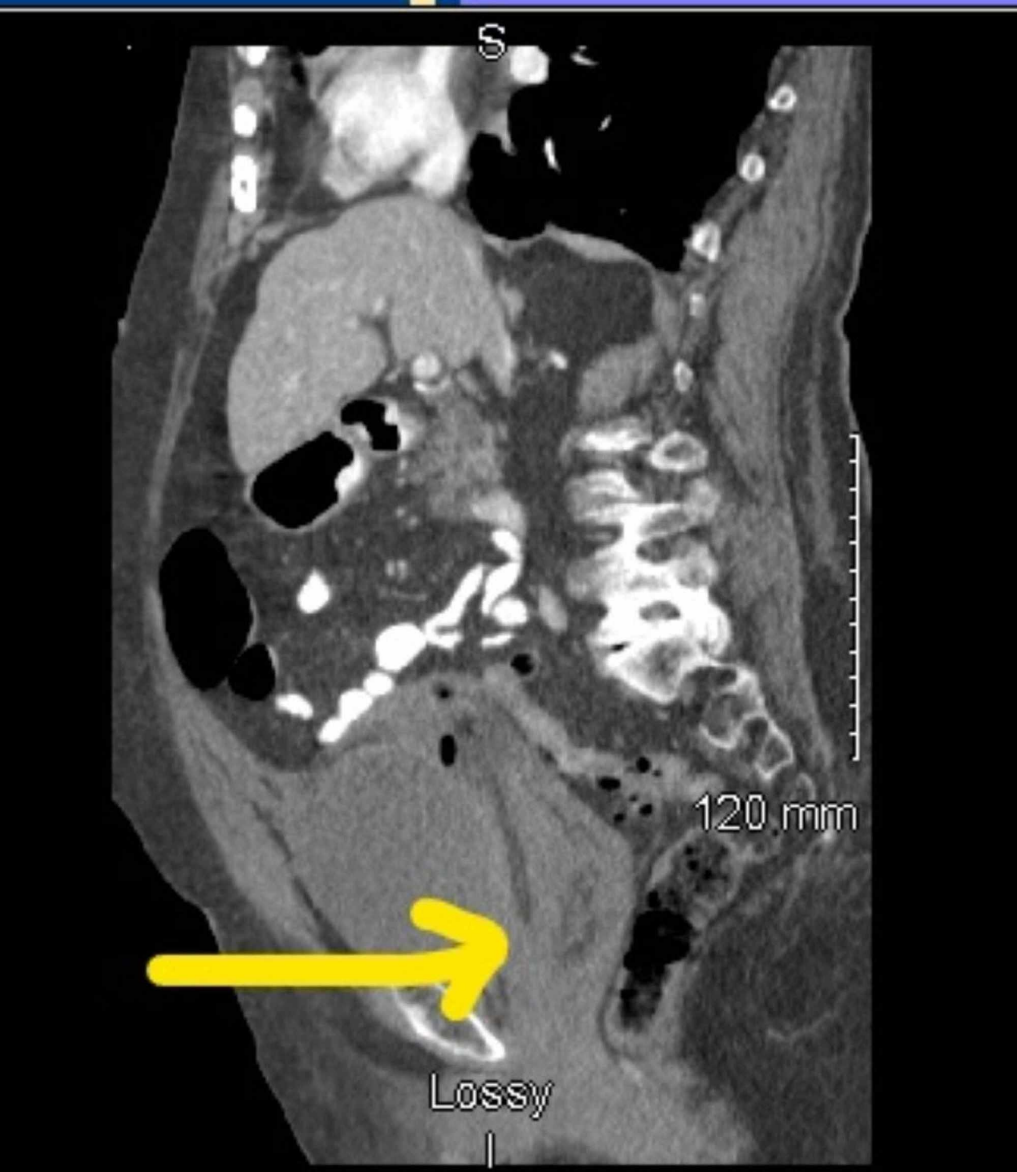
A large rectus sheath hematoma compressing on bladder neck

A 93-year-old woman presented to the emergency room with complaints of inability to pass urine over the course of one day. The patient was a weak debilitated woman, residing in an assisted living facility with a Karnofsky Performance Index of 40 (disabled, requires special care and help). Her past medical history was significant for chronic obstructive pulmonary disease, hypertension, hyperlipidemia, and atrial fibrillation needing oral Coumadin. On examination in the emergency room, the patient's vital signs were within normal limits, and the only history provided was the patient had not passed urine for a day by the patient's care provider. Clinical examination was positive for a mass palpable in the abdomen which was globular and firm in consistency. A routine set of blood work at the time was remarkable for anemia with a hemoglobin of 6 g/dL and an elevated INR to 4.5. A computed tomography scan of the abdomen and the pelvis at the time of admission with intravenous contrast revealed a large grade 3 RSH extending into the pelvis and causing compressive symptoms over the bladder outlet (Figures 1, 2). There was no evidence of contrast extravasation in the hematoma. The patient was immediately transfused packed red blood cells to maintain a hemoglobin of more than 8 g/dL. Coumadin was held and INR reversed with the help of fresh frozen plasma and vitamin K. The patient had a Foley catheter placed to relieve bladder outlet obstruction. Over the subsequent day, the patient continued to have stable hemoglobin and make urine. In view of the patient’s poor performance index and high risk for surgery, a 14 French catheter was placed in the hematoma. Over the subsequent days, clinical examination revealed a reduction in the size of the abdominal hematoma and the Foley catheter was removed on four days post procedure. The patient was able to void independently. The catheter was removed and the woman was discharged from the hospital. At the two-week period, she continued to have a swelling which was smaller in size, however, she remained asymptomatic from it.

## Discussion

A rectus sheath hematoma (RSH) is the result of an accumulation of blood in the sheath of the rectus abdominus muscle, typically caused by epigastric artery rupture or muscular tear [1-5]. The rupture may be caused by external trauma to the abdominal wall, iatrogenic trauma from surgery, or the rectus muscle vigorously contracting from coughing, vomiting, or straining at the stool [1-6]. Hematomas originating below the arcuate line are caused by damage to the inferior epigastric artery and its branches [7-10]. These hematomas are more prone to bleed, and are more likely to shift across the midline descending into prevesicular space [9-11].

Anticoagulants are associated with the primary cause of rectus sheath hematomas [2-4]. Other predisposing and contributing factors to the development of RSH include obesity, corticosteroid therapy, arterial hypertension, pregnancy, previous abdominal surgeries, and thrombophilia [1-5].

There are three types of RSH based on CT scan findings [9-11]. If the hematoma is intramuscular as well as unilateral and does not dissect along the fascial planes, it is classified as type 1. Type 2 hematomas are intramuscular with blood between the muscle and the transversalis fascia, can be unilateral but are usually bilateral, and no blood is found in the prevesical space. With type 3 hematomas, as discussed in this case, blood is seen between the transversalis fascia and the muscle, in the peritoneum, which can dissect into prevesical space [2-11]. Unlike types 1 and 2, type 3 has a higher prevalence of a hematocrit effect, requiring blood transfusion as there is no sheath below the arcuate line to contain the hematoma and this reduces the compressive force of the sheath [6-11]. With all three types, only in rare cases of hemodynamic instability, such as failure of fresh frozen plasma (FFP) and fluid resuscitation, is surgical intervention required [3-11].

The majority of cases of RSH are treated non-surgically [1-5]. An initial conservative approach involves the removal of predisposing factors, the use of blood transfusions, coagulation correction, icing, external hematoma compression, pain management, observation, and blood pressure regulation [1-6]. Persistent hemodynamic instability and abdominal compartment syndrome require management using an invasive approach. The current invasive treatment of choice is therapeutic angiography with embolization of the bleeding vessel [5-11]. Other methods of intervention include operative therapy with hematoma evacuation, ligation of bleeding vessels, and closed-suction drainage [5,9-11].

As there are no prognostic factors that can predict hemodynamic instability, frequent CT monitoring is necessary for RSH to determine signs of progression [5-10]. This is especially true with regard to type 3 RSHs, as mortality rates can be as high as 28.6% as shown in a retrospective study [5,6,9]. In general, however, RSHs are mostly self-limiting and conservative treatments are successful in most cases [1-5]. More aggressive approaches may be required with type 3 as rupture and intraperitoneal bleeding are more common [9-11]. These higher morbidity strategies involve dissection into hematoma, evacuation of the clot, and direct control of bleeding vessels. Inferior epigastric vessel ligation or angioembolisation using interventional radiology may also be needed [4,6,9,11]. In some instances such as in this case, based on individual clinical assessment, a type 3 RSH hematoma was managed successfully and effectively through a conservative approach by using percutaneous drainage. This allowed the body to absorb the remaining hematoma and for faster recovery time.

## Conclusions

Grade 3 RSH is an unusual condition, which extends to cause bladder outlet obstruction. A small subset of high-risk patients can be managed non-surgically by relieving compressive symptoms using a percutaneous drain and then allow the hematoma to shrink gradually.
